# A temporal hierarchical feedforward model explains both the time and the accuracy of object recognition

**DOI:** 10.1038/s41598-021-85198-2

**Published:** 2021-03-11

**Authors:** Hamed Heidari-Gorji, Reza Ebrahimpour, Sajjad Zabbah

**Affiliations:** 1 Faculty of Computer Engineering, Shahid Rajaee Teacher Training University, P.O. Box 16785-163, Tehran, Iran; 2 School of Cognitive Sciences, Institute for Research in Fundamental Sciences (IPM), P.O. Box 19395-5746, Tehran, Iran; 3 Department of Psychology, Justus Liebig University Giessen, Giessen, Germany

**Keywords:** Cognitive neuroscience, Computational neuroscience, Visual system

## Abstract

Brain can recognize different objects as ones it has previously experienced. The recognition accuracy and its processing time depend on different stimulus properties such as the viewing conditions, the noise levels, etc. Recognition accuracy can be explained well by different models. However, most models paid no attention to the processing time, and the ones which do, are not biologically plausible. By modifying a hierarchical spiking neural network (spiking HMAX), the input stimulus is represented temporally within the spike trains. Then, by coupling the modified spiking HMAX model, with an accumulation-to-bound decision-making model, the generated spikes are accumulated over time. The input category is determined as soon as the firing rates of accumulators reaches a threshold (decision bound). The proposed object recognition model accounts for both recognition time and accuracy. Results show that not only does the model follow human accuracy in a psychophysical task better than the well-known non-temporal models, but also it predicts human response time in each choice. Results provide enough evidence that the temporal representation of features is informative, since it can improve the accuracy of a biologically plausible decision maker over time. In addition, the decision bound is able to adjust the speed-accuracy trade-off in different object recognition tasks.

## Introduction

Recognition of a wide variety of the visual objects and exploiting the visually obtained information for making different decisions, are not only about the human brain^1,2^ ability, but also about the brains of much less developed creatures, like rodents^3,4^. Studies on the mechanism of this fundamental capability have shown that the process of object recognition in both representation^5^ and decision levels^6^ takes different time for different objects. It takes longer for the noisier objects to be represented in the inferior temporal cortex (IT)^7^ while the decisions to recognize them are also slower and less accurate than those which are less noisy^8,9^. Importantly, the accuracy and the time of the recognition in the brain can vary due to different regimes of the speed-accuracy trade-off^10^. Thus, a reliable explanation for the underlying mechanisms of object recognition in the brain should be able to explain both the accuracy and the speed of this process. However, computational models of object recognition have usually ignored the brain’s dynamics at the representation and the decision-making levels^11–19^ such that most of them are only capable of following either the accuracy^11–13,15,16,19^ or the reaction time^20^ of recognition.

One of the first hierarchical cortex-based models of object recognition, compatible with the available neurophysiological evidence, is the model Neocognitron^21^. Neurons in primary layers of this model are sensitive to simple stimuli such as lines in specific angles. Next, neurons in the subsequent layers, combine the outputs of the previous layers with each other to respond to more complex stimuli. HMAX is another model with a structure similar to Neocognitron, but with different combination rules. This model succeeded in producing a comparable performance to human behavior in an animal versus non-animal object recognition task^22^. Further modified versions of HMAX were also presented in other studies later, to explain the mechanisms of object recognition in different tasks^16,18,19,23^. In 2007, Masquelier and Thorpe modified HMAX using spiking neural networks and the STDP learning rule^12^. Their model performs better in object categorization compared with the original HMAX model, and more importantly, it explains the process of object recognition in the brain with a more biologically plausible structure.

In all the above-mentioned studies, biologically plausible processes, which are used to extract informative features from the input stimuli, finally form a static representation. Then a non-biological classifier, such as a support vector machine or a radial basis function, uses the extracted features to determine the input category. These models can follow human choice accuracy in rapid categorization tasks. However, they do not explain the human response time which is an important aspect of behavioral responses^24^ and more importantly, is in the trade-off with the accuracy (spending more time improves the accuracy)^25,26^. On the other hand, the model presented by Mirzaei et al.^20^ explains the response time of observers in a rapid recognition task, but it cannot explain the accuracy of choice and its relation to the reaction time.

Here, we present a temporal hierarchical feedforward model in order to explain the accuracy as well as the reaction time in a rapid object categorization task. The input stimuli are represented temporally by spikes that are generated in a hierarchical feedforward structure which simulates the ventral pathway in the brain. Generated spikes are then transferred to a decision-making layer. This layer contains units that accumulate spikes (as temporal evidence) over time in support of possible choices. The input category will be recognized as soon as an accumulator reaches a threshold. This accumulation to the bound mechanism of decision making is a well-known biologically plausible decision-making model^2,27–30^. Results show that the temporal representation of the input stimuli is informative, since it can explain human reaction time as well as the accuracy, in an object recognition task. Importantly, the accuracy improves by increasing the decision threshold, supporting that the information represented over time is not redundant and thus can well explain the speed-accuracy trade-off. The model can shed light on the mechanism of object recognition in the brain, from the level of stimulus representation up to the behavior.

## Materials and methods

In this section, we first give details about the spiking HMAX model proposed by Masquelier and Thorpe in 2007, as our base model^12^. Next, our proposed model will be introduced and we will show how the behavioral results of a psychophysics task are employed for adjusting the model parameters.

### The base model

HMAX and Spiking HMAX are plausible object recognition models of the brain with simple feedforward and hierarchical architectures, corresponding to different layers of the ventral stream of the visual pathway in the brain. Unlike the HMAX, the spiking HMAX considers neuronal constraints. It uses integrate-and-fire (I&F) neurons with a simple STDP training method. The I&F model of a neuron assumes that, when an input current is applied, the membrane voltage increases until it reaches a constant threshold and thus generates a single spike. Through unsupervised STDP, intermediate-level neurons become selective to the frequent patterns in presented images. Over time, responses get more reliable and faster and their latencies decrease. This hierarchical network consists of four layers (S1, C1, S2, and C2) which are briefly explained here.

#### S1 layer

S1 units correspond to the simple edge-detector cells of the primary visual cortex (V1) as described by Hubel and Wiesel^31^. In fact, application of a convolution operator, enables the S1 units of the model to detect the edges in the input image. The convolution matrices are 5 × 5 in size and approximately similar to the Gabor filters of wavelength 5 and width 2. These kernels are available in 4 different angles; 0 + 22.5, 45 + 22.5, 90 + 22.5, and 135 + 22.5 in degrees (the added rotation is to prevent focusing on the horizontal and vertical edges). These filters are applied across five different sizes of the input image (100%, 71%, 50%, 35%, and 25%) and overall, the S1′s output will be composed of 4 × 5 = 20 images.

#### C1 layer

C1 functionally corresponds to the complex cells of the visual system. In this layer, a 7 × 7 window is max-pooled and then slid ahead with an overlap value of unity for each of the 20 images outputted from S1. The resulting size will be 1/6 of the S1′s inputs. For example, for an original S1′s input of size 454 × 300, the output would be 5 images of sizes: 75 × 50, 53 × 35, 38 × 25, 26 × 17, and 19 × 12 for each orientation angle, while the overall number of representation maps would be, still 20.

There is another max-pooling mechanism in C1, for images of the same sizes, each belonging to one of the 4 orientations calculated by S1. The max-pooling in this next stage is done by taking the pixelwise maximum of the 4 m × n images and storing the result in a new m × n matrix.

At this stage in C1, there are representational maps of the original image, in 5 different sizes. Then spike times are calculated as reciprocals of the points within each of these matrices. The position of the point generating the spike and also the related scales and rotations are stored along with the spike times to be utilized in next layers. By sorting the vectors containing this information according to the spike time, a spike train will be generated to be fed to the following layers. This spike generation method is supported by empirical studies of V1 in the brain, indicating a relationship between the stimulus contrast and the time of spikes.

#### S2 layer

Cells in S2 correspond to the intermediate visual features. In this study, our S2 layer contains of 20 cells. We have trained ten cells of the S2 layer for detection of the house category and ten other cells for the face category. Each cell consists of 5 maps and each map matches in size to the corresponding C1 map. Spikes coming from C1 affect only the corresponding map with the same scale. If any point within any of the containing maps of a cell reaches the threshold, a spike will be sent to C2.

#### Our modification on S2 layer

For the training phase of this layer, we utilized the same simple STDP learning method as in the base model. However, after the training phase, we decreased the firing threshold of S2 neurons. Unlike the base model which has one-winner-take-all mechanism, our model resets the potential of S2 neurons after each spike and allows them to reach the threshold for an unlimited number of times. With this modification, the model has a more brain plausible mechanism in comparison with the neurons that can only generate a single spike.

#### C2 layer

Whenever the threshold is hit for S2 map, the neurons of this layer will fire and the classifier would use the timings of the first spike of these neurons to classify the input image. Due to our modification on the layer S2 of the model, reducing the threshold and allowing the neurons to fire more than once, there are spike trains, instead of single spikes, that reach the C2.

Finally, similar to other brain plausible object recognition models, spiking HMAX uses some non-biological classifiers to classify the input images based on the extracted features. We also substituted the classifier layer in the spiking HMAX model with a biological decision-making mechanism in order to better explain the human behavior.

### The proposed model

**Figure 1:**
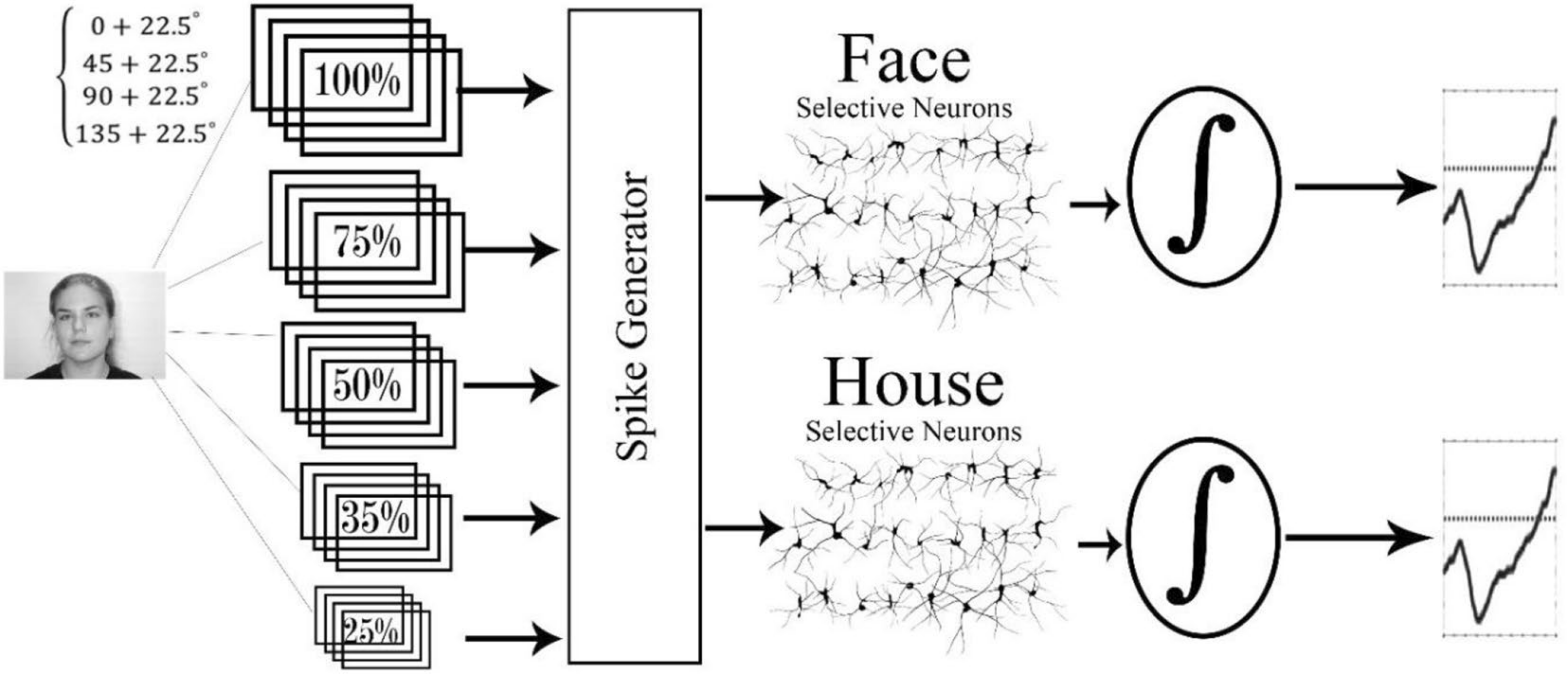
The image enters the model with different scales and orientations. Then the edge detection and maximization steps are performed and the spike data is generated. In the following steps, the spikes are fed into the face selective and house selective neurons. The output of these neurons is sent to the decision-making layer. If the neuron reaches the threshold of the face (house) category, images are categorized as face (house).

The proposed model, similar to other brain plausible object recognition models, consists of two main parts; the feature extraction part, which corresponds to the ventral stream, and the decision-making part, which corresponds to the decision-making area in the brain (Fig. 1, see also supplementary Fig. S1 for a detailed diagram of the full model).

#### Feature extraction part (temporal model of ventral stream)

In the feature extraction segment, we modified the spiking HMAX model in a way that neurons can generate an unlimited number of spikes in the S2 layer. Then, instead of sending only the first spike, a train of spikes will be sent to the next layers. Thus, the proposed mechanism can generate spikes over time and provides the model with the ability of forming a temporal representation of the input features. In the decision-making segment on the other hand, there are two simple accumulators (corresponds to two different classes of input images; face and house) each of which accumulates spikes received from neurons of the previous layer. In the following, both segments of the model are explained in detail.

**Figure 2:**
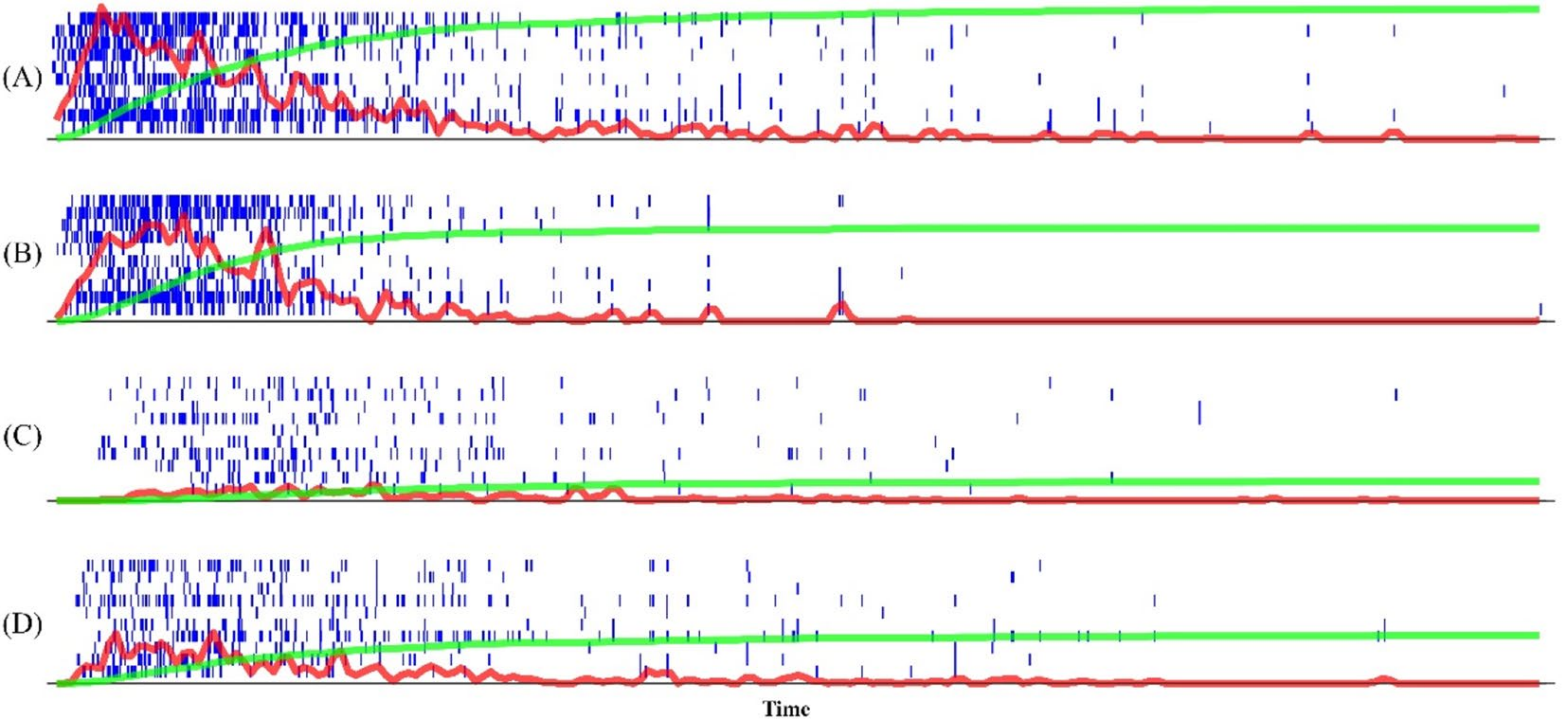
Model raster plot for 10 face-selective neurons. Red curve indicates running average firing rate (AFR) of model neurons. Cumulative AFR is represented by green. (**A**) face without noise; (**B**) face with 20% noise; (**C**) face with 30% noise; (**D**) house without noise. Neurons in all four panels are the same STDP face trained neurons and only the input image is different.

The first segment of our model is based on the spiking HMAX model, as described in the previous section, but with some modifications. First, we trained spiking HMAX twice without any changes to the base model, one time with face and another time with the house dataset. This pre-trained stage of the proposed model is to make the face, and house-selective neurons, able to represent face and house features. The training mechanism is well-explained in the previous literature^12^. Face and house-selective neurons generate a single spike if the input pattern is similar to what they have learned during the training phase (Fig. 1). Afterwards, we reduced the selective neurons’ threshold in order to increase their tolerance to the input variability. This way, neurons fire not only to the exact patterns they learned, but also to other similar patterns. Raster plots in Fig. 2 show spikes which are generated by face neurons in response to noisy images. While these neurons were trained by noiseless face images, they generated many spikes in response to noisy face and noiseless house images however the number of generated spikes depends on the level of the noise and the content of the image.

**Figure 3:**
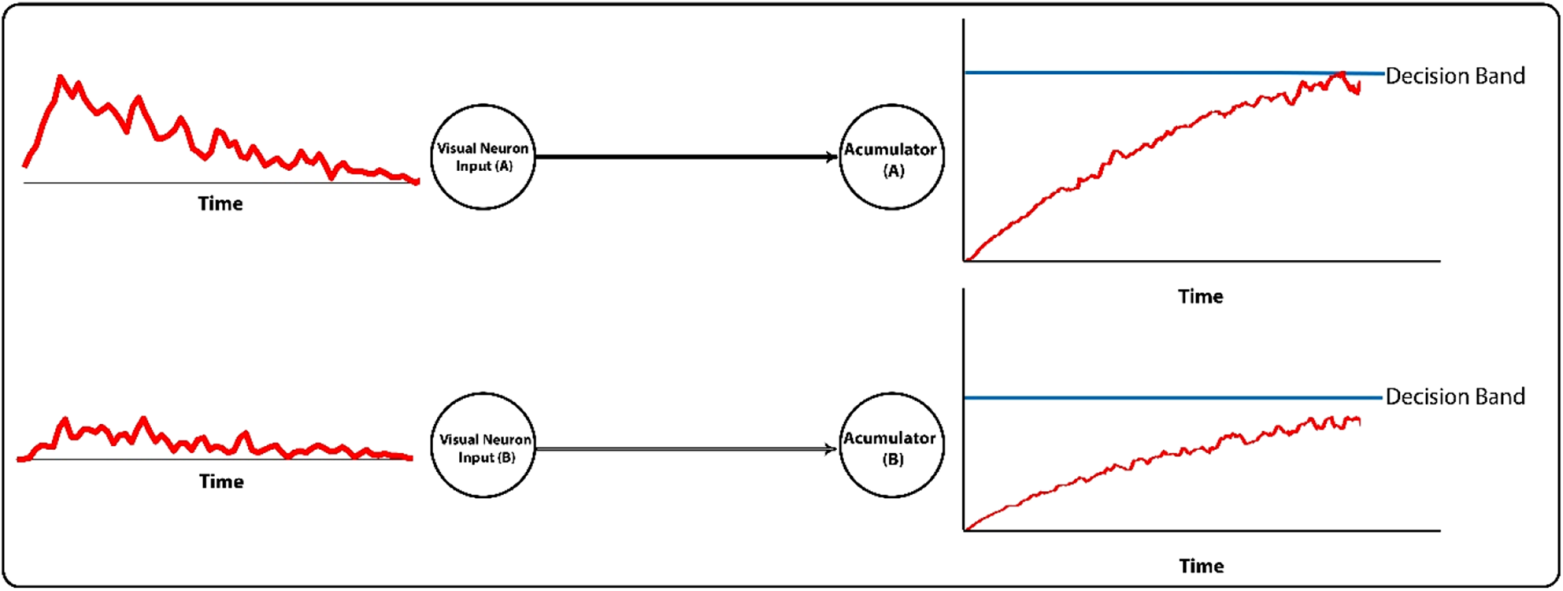
The schematic of accumulators in decision-making layer. Spikes generated by face and house selective neurons in the visual pathway model are entered to the corresponding accumulator. Spikes are integrated over time until they reach the threshold, and then the decision is made. The accumulator that reaches faster to the threshold identifies the class of input image. Decision time is calculated from the start of information accumulation until reaching the threshold.

The second segment of the model is responsible for making decisions based on spikes received from the house-selective and face-selective neurons. Our proposed model for making decision based on the generated spikes in the preceding layer that represents the stimuli features, stems from a very well-known model of decision making in the brain which is called the Accumulation-to-bound-model. According to this model, the brain accumulates information toward different potential choices and makes its final decision as soon as the accumulated information for an option touches a threshold. There are many studies showing that the firing rate of neurons in regions involved in decision making, such as DL-PFC, FEF, LIP, and SC in the brain exhibits a ramping activity^2,27^ before making a decision and reaches a stereotype of activity at the time of making a choice, which supports the “accumulation of information toward a bound” hypothesis. Relying on such evidence, we have used an accumulation-to-bound operation as the decision-making part of our proposed, biologically plausible, object recognition model. There are two accumulators in the decision-making segment of the model; one accumulates spikes generated by all of the face-selective neurons and the other accumulates spikes generated by all of the house-selective ones. Green curves in Fig. 2 show the accumulated spikes over time. The slope of these curves depends on the level of the noise in the input image and whether the image is face or house. These integrators, as shown in Fig. 3, accumulate the spikes until they hit the decision threshold. The choice is defined by the accumulator that first touches the threshold. Another training step is also required to set the threshold levels and other parameters of decision-making apparatus.

#### Decision-making model components

Spikes generated in face and house selective neurons which are equivalent to face and house selective regions in the cortex^32,33^ are being accumulated to reach a bound. The accumulator which earlier reaches the threshold is the so-called winner and the time spent over accumulation phase in the subthreshold regime is considered as the decision time. However, for the simulation of human reaction time, the decision time is added to a constant time that corresponds to the temporal duration of the motor command transmission (Eq. 2). This value is obtained automatically in the second training phase where we optimize the parameters of the decision-making layer. The error function of this optimization is defined in Eq. 1.

1 $$MSE = \mathop \sum \limits_{i = 1}^{10} \sqrt {\left( {RT_{{face\_model_{i} }} - RT_{{face\_psycho_{i} }} } \right)^{2} + \left( {RT_{{house\_model_{i} }} - RT_{{house\_psycho_{i} }} } \right)^{2} }$$

where $RT_{{face\_psycho_{i} }}$ and $RT_{{house\_psycho_{i} }}$ are the average reaction time for the images of house and face in the noise level “i” during the psychophysics test, respectively. To calculate $RT_{{X\_model_{i} }}$ $\left( {X = face\;or\;house} \right)$ we used the below equation.

2 $$RT_{{X\_model_{i} }} = a \times rt_{{X_{i} }} + RT_{motor}$$

where $RT_{{X\_model_{i} }}$ is the reaction time of the model when the stimulus in noise level i is categorized as x (x = face or house); $\alpha$ is the time scaling parameter; $rt_{{X_{i} }}$ is the time that it takes the accumulator x to touch its bound when stimulus is in noise level i (Eqs. 3 and 4), and RT_Motor_ is the required time for motor command transmission. To calculate the threshold in face and house accumulators ($TH_{face}$ and $TH_{house}$ respectively), RT_Motor_ and $\alpha$, the MSE from (1) is minimized by using SIMPLEX^34^ method (implemented by the fminsearch function in Matlab 9.0 (MATLAB, 2016. version 9.0 (R2016a), Natick, Massachusetts: The MathWorks Inc.)) which is a direct search method that has been widely used in unconstrained optimization problems^34^.

3 $$rt_{X} = t if AC_{face} \left( t \right) = = TH_{face} and AC_{house} \left( t \right) < TH_{house}$$

4 $$rt_{X} = t if AC_{face} \left( t \right) < TH_{face} and AC_{house} \left( t \right) = = TH_{house}$$

$TH_{face}$ and $TH_{house}$ are the face and house threshold respectively, and $AC_{face}$ and $AC_{house}$ are defined as below:

5 $$AC_{face} = \mathop \sum \limits_{t} \left[ {v_{face} \left( t \right) - u \cdot v_{house} \left( t \right)} \right]$$

6 $$AC_{house} = \mathop \sum \limits_{t} \left[ {v_{house} \left( t \right) - u \cdot v_{Face} \left( t \right)} \right]$$

where $AC_{face}$ and $AC_{house}$ are the firing rates of the face and house accumulators respectively. These firing rates are functionally the integral of spikes generated by the face-selective and house-selective neurons, respectively. $v_{face} \left( t \right)$ is the input information value at time t, from the face-selective population of neurons (same for house-accumulator), and u is a coefficient for the opposing accumulator, which is set to zero in our computations.

Thus, face threshold, house threshold, the model time scale, and non-decision time are four free parameters of the decision-making layer. It should be noted that all free parameters are the same for all input images regardless of their difficulty (noise level).

### Psychophysics test

10 subjects (7 men and 3 women, aged between 20 and 35 years) participated in the psychophysical test. All participants were in normal state regarding their visual acuity and had no familiarity with the images used during the test, based on their declaration. The participants were seated on a fixed chair with a constant distance from the monitor in a dark room. The distance between the participants’ eyes and the monitor was set to 59 cm and a chinrest was used to ensure that the distance is maintained throughout the experiment. The display screen was a CRT type with a resolution of 600 × 800 pixels.

### Datasets

#### Face and house

We used 200 images from Caltech 101^35^ dataset with equal numbers (100) for face and house classes to compare the model with human behavior. All the images were 300 × 450 pixels (7 × 7°) and also gray scaled with 8-bit color code. Each figure was used in 10 different noise levels, and in total, 2000 images were generated for the test. Every participant responded to one block of samples containing 200 images (100 human faces and 100 houses, with 10 images in each level of noise for house or face). Each image was presented only once in each noise level. Participants could see the image only once during the test and there was no chance for getting familiar with the images. The Psychophysics Toolbox.V3 of MATLAB^36^ was used to design the task.

#### Leopard, butterfly and motorbike

We also employed leopard (200 images), butterfly (91 images) and motorbike (200 images) images from Caltech 101^35^ to examine behavior of the model in the absence of artificial noise. Keeping the aspect ratio of images (using zero padding when necessary), all images were resized to 300 × 450 pixels and also gray scaled with 8-bit color code.

### Noise production

**Figure 4:**
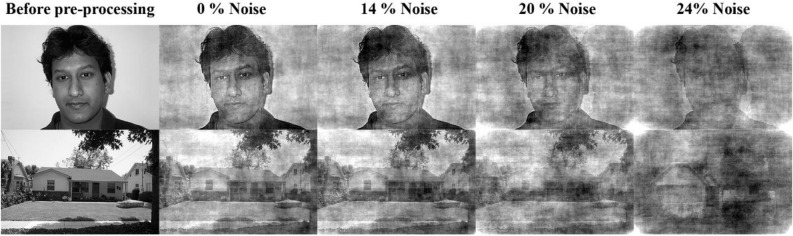
The sample of used images in the model and psychophysical test. The upper row is face and the lower row is house with different noise levels.

For every image of face-house dataset, a 2-D Fourier transform was taken and the phase and magnitude were obtained. The average magnitude of all images was also computed. The noisy phase is a weighted (corresponding to required level of noise) linear combination of the original phase and a phase noise between − π and π. Finally, the noisy image is obtained from the inverse Fourier transform of the average of magnitude and noisy phase of the main image (Fig. 4)^37^.

**Figure 5:**
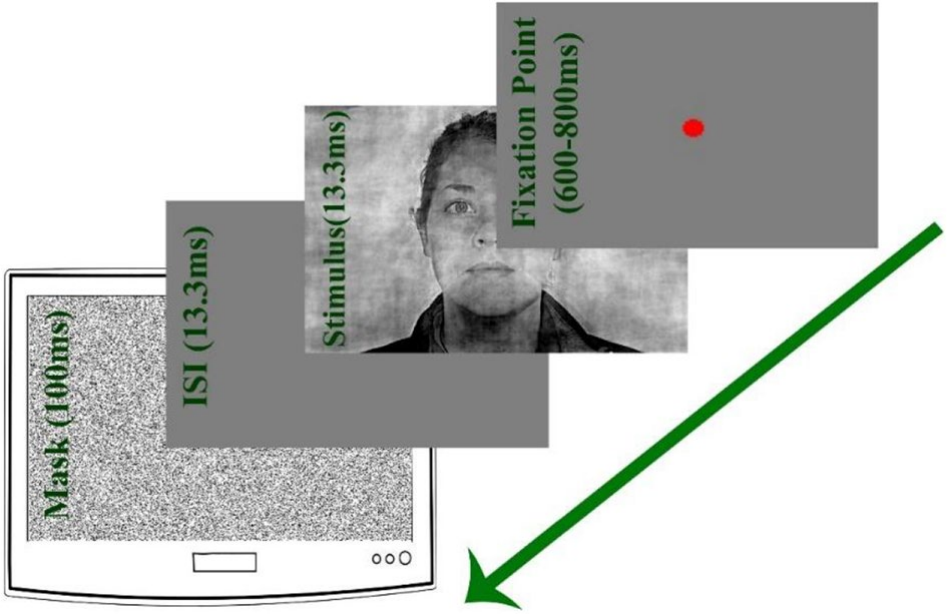
Psychophysics paradigm. Each trial starts by 600–800 ms presentation of a red dot in the center. The stimulus and a blank gray page will be both presented for 13.3 ms sequentially. The trial will be finished by presentation of a noisy mask for 100 ms.

In all trials, at the beginning of the test, a fixation point in the center of the monitor was shown for a random period between 600 and 800 ms. Then, an image from the dataset (containing 200 images) was shown randomly for 13.3 ms on the monitor. The presented image is a house or a face in one of the 10 noise levels. Then, a gray screen is shown for 13.3 ms as an Inter stimulus interval (ISI). Afterwards, a noisy mask was presented for 100 ms in order to block the recurrent activities in the brain (Fig. 5). Subjects reported face and house by pressing F and H keys on the keyboard, respectively, as fast and accurate as they could. The reaction time from this behavioral test was used to set the free parameters of Eq. (2).

### Ethics approval

All experimental procedures were confirmed to guidelines determined by the 1964 Helsinki declaration and were approved by the ethical committee of Shahid Rajaee University. This article does not contain any studies with animals performed by any of the authors.

### Informed consent

Informed consent was obtained from all individual participants included in the study.

## Results

The proposed model contains layers corresponding to the visual pathway, and a layer corresponding to the decision-making area in the brain. Once trained, neurons within the visual pathway layers of the model become selective to the features of house and face in the input image. Depending on the presence and the strength of features in a given input stimulus, neurons in the last layer of this pathway generate spike trains. Face (house) related information represented in the neural activity of the face-selective (or house-selective) neurons is then accumulated over time by an accumulator in the decision-making layer. Upon reaching a decision threshold, the model decides whether the input image is a face or a house. It should be emphasized that there were not two separate pathways for different categories (face-house) of input images to be represented by the model. Therefore, some features in a face image may activate or increase the activation of house-selective neurons, which consequently increase the activity of the house-accumulator, and vice versa. Each accumulator which touches the threshold earlier, determines the model’s final decision, and a linear scale of the threshold crossing time determines the decision time. The final response time of the model is the summation of the decision time and a constant value corresponds to the non-decision time. Free parameters of the decision layer were optimized in a way that the model maximizes the likelihood of generating the subjects’ behavior in the psychophysical task (See supplementary Table 1 for optimized parameters). Then, the performance and the response time of the proposed model were compared with those of humans. Moreover, we investigated whether the temporal representation of stimuli improves the performance compared to the spiking HMAX as a biologically plausible model with static representation, and some well-known deep neural networks. Finally, the temporal information of the model was investigated through some computational experiments to show how informative they are.

### Firing rate of selective neurons depends on the category and the strength of the stimulus

Figure 2 shows spikes of ten face-selective neurons (raster plots) for a face image without noise (panel A), a face image with 20% noise (panel B), a face image with 30% noise (panel C) and a house image without noise (panel D). The raster plot becomes denser as the stimulus noise decreases. This effect comes from the fact that the model is sensitive to edges in its first layer (S1) and thus generates lower (higher) values in the output of this layer when edges disappear due to the high (low) level of noise in the input image. A higher value in S1 causes a faster spike in C1. In addition, neurons in S2 will faster reach their threshold when: 1—they receive enough inputs from C1 and 2—the input stimulus contains features which are closer to previously learned features in this layer. Therefore, Fig. 2 indicates that: 1—edges are sharper in the low noise images 2—the representative patterns which were learned by the face-selective neurons are clearer in the stimulus without noise than in the noisy one. Results are similar for the house-selective neurons.

### The proposed model can explain both the performance and reaction time of human observers

**Figure 6:**
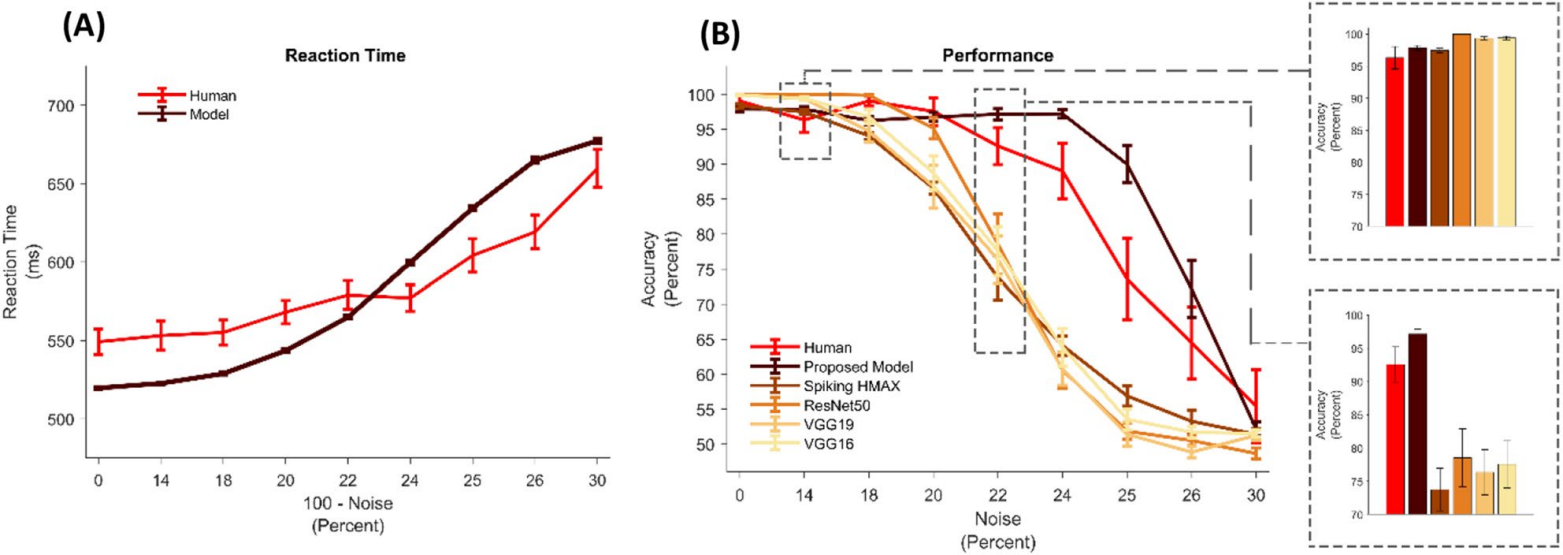
Human versus models’ behavior. (**A**) Reaction time of the human and the proposed model. The light red curve shows the human reaction time in the psychophysical experiment with different noisy images and the dark red one shows the reaction time of the model for the same images. (**B**) Performance of human and different models. The darkest curve represents the average performance of 10 people for 20 stimuli in each noise level (10 face stimuli and 10 house stimuli). The light red curve is the proposed model and the others are spiking HMAX and three different versions of deep models. Performance of each model is the average of 10 runs using n-leave out method (n = 10). Bar graphs emphasize the performance in specific noise levels (14 and 24 percent). Error bars are SEM.

The model response time to report a decision about input stimuli in different levels of noise can follow the trend of human reaction time in those levels (see Fig. 6A). Each point in the figure is the average of response times for 20 different images from house and face categories presented to participants and the model. The light red curve shows the average and the standard error of the participants' reaction times at each noise level. The dark red curve presents the average response time of the model in 10 runs. To make the model independent of the training data the n-leave out method was used (n = 10). Since the model generates spikes with different rates, depending on the noise level in the input image (as shown in Fig. 2), we expect to see that the corresponding accumulated spikes touch the decision threshold in different times.

It is important to note that although the visual pathway layers of the proposed model were learned only by noiseless stimuli, the model can well follow human performance in noisy stimuli (Fig. 6B). Figure 6B also shows that the proposed model performs better than classic spiking HMAX, which only uses the first spike with a non-biologically plausible classifier. This indicates that although the first spike (classic spiking HMAX) can solve the object recognition problem in noiseless conditions, as the brain does, in the noisy condition the model needs more spikes to be able to perform as well as a human does.

### The lack of temporal knowledge was not compensated by using deep neural networks

The lack of temporal knowledge was not compensated by using deep neural networks which are considered as more powerful feature extractors. As illustrated by the different colors in Fig. 6B, several well-known deep models, including VGG16^38^, VGG19^38^, and ResNet50^39^, were used. Models were pre-trained with ImageNet^40^, and through a transfer learning method the last layer of these models with 1000 neurons (number of categories in ImageNet) was replaced by a layer with two neurons (for house and face). During the training procedure, using Adam optimizer, only weights of the two last layers were adjusted. As expected, the performance of deep models is almost perfect (100%) on noiseless or low-noise images (some are better than human), but as the level of noise increases, it drops much faster than the human’s performance and also than our proposed model. These results suggest that spikes generated in the visual pathway layers of the proposed model are informative in the way that a simple structure of spike accumulation up to threshold as a decision model can better explain human behavior than more complex models of feature extraction and decision making.

### Generated spikes convey information about the stimuli classes in all noise levels

**Figure 7:**
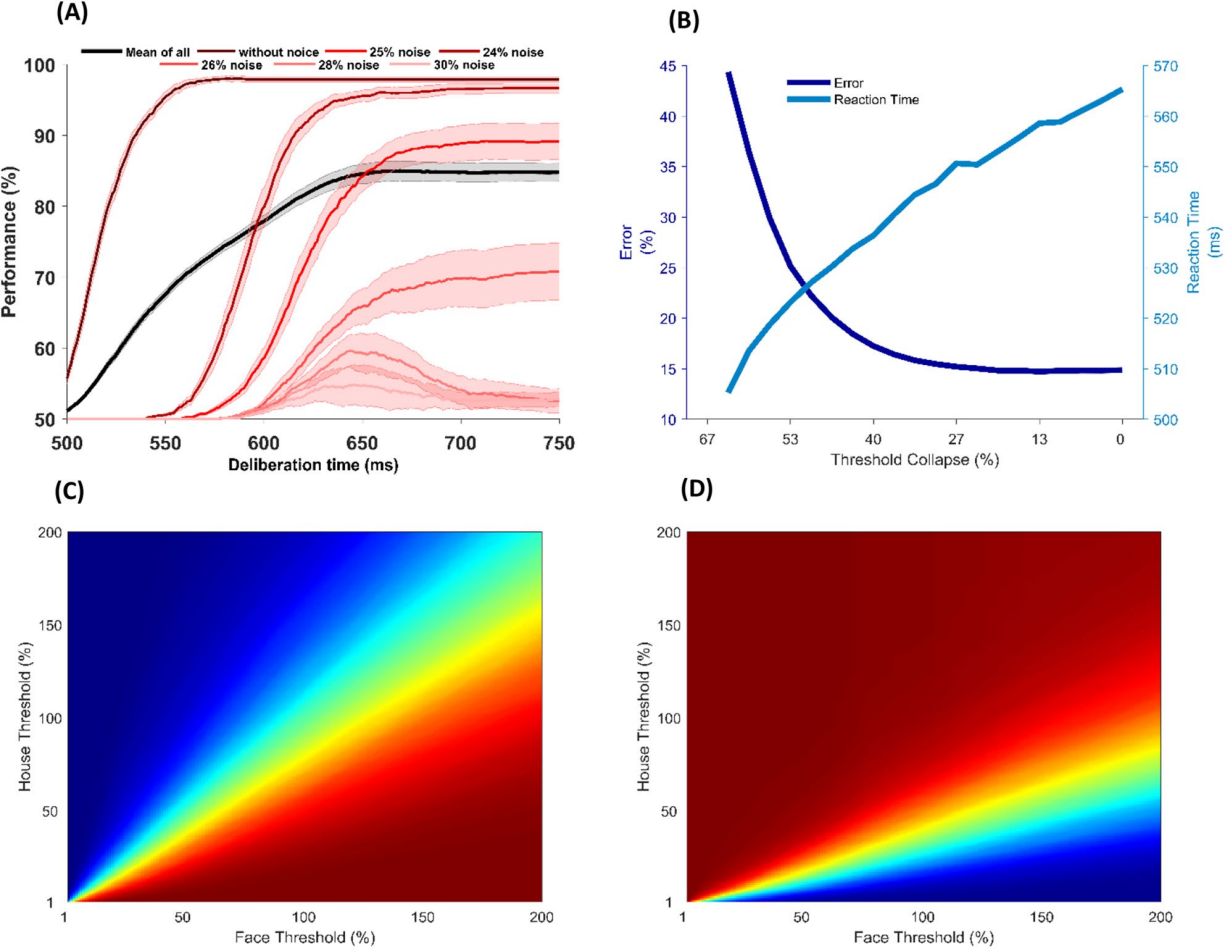
Temporal information is informative. (**A**) Having more time for information accumulation improves the performance. X-axis indicates the time that the model is allowed to accumulate spikes (deliberation time). Y-axis shows the performance of the model. The darker the red color, the less the noise level. The black color is the average of performance in all levels of noise. (**B**) Speed-accuracy trade-off can be controlled by threshold. The X-axis indicates the percentage of collapse in the decision bound. The left y-axis shows the error rate (dark blue curve) and the right y-axis shows the reaction time of the model. (**C**) Performance of face choices in different face and house threshold for noise level 26%. Warmer colors show a higher performance in face choices of the model. (**D**) Performance of house choices in different face and house threshold noise level 26%. Warmer colors show a higher performance in house choices of the model.

Masquelier et al. showed that the time of the first spike is sufficient for the rapid categorization of a simple and noiseless stimulus, however, as shown in Fig. 6B, is not sufficient for the categorization of the noisy stimulus with the same accuracy as humans can perform. On the other hand, the accumulation of other spikes toward a decision bound improves decisions to the range of human accuracy. However, one may speculate that the improvement of the accuracy in the temporal model (proposed model) is not due to the temporal information, but might be the result of using a different decision-making model. In order to rule out this hypothesis and investigate whether the spikes generated by the visual pathway layers of the model are informative or redundant, performance of the model was calculated over time. Figure 7A shows that letting the model to use more information over time will increase the categorization performance. In other words, spikes generated in different time points which are indicators of the edge’s strength and the strength of the pre-learned patterns in the input image, are not redundant and they can improve the categorization performance. This improvement in performance as a result of longer stimulus duration, is in line with results in behavioral studies^29^. It should be noted that, in this analysis, crossing the bound cannot be the decision rule because we forced the model to decide about input stimulus at different time points, no matter if any of the accumulators cross the bound or not. Thus, here, the decision is determined by the accumulator that is closer to the bound. It is also noticeable that, for the very noisy input stimuli, increasing the time of processing does not always improve the performance monotonically, because in these stimuli, information is hardly reliable, and spikes generated late in the model are induced by noise.

### Decision threshold of the model can represent the speed-accuracy trade-off

In the accumulation-to-bound model, the decision bound determines the speed accuracy trade-off, i.e., increasing the decision threshold is equivalent to making slower but more accurate decision. Therefore, in paradigms that emphasize on accuracy, the decision threshold is more than that in the paradigms which more emphasize on speed of making a decision^10,25,26^. In order to investigate whether the decision bound in our decision-making layer can truly play the role of a speed-accuracy trade-off, we investigated the performance and reaction time of the model in different threshold levels. Figure 7B shows the effect of the variation of the decision bound on the performance (dark blue) and reaction time (light blue) of the model. The x-axis represents the 200 threshold values, and the left and the right y-axis stand for the average performance and response time of the model in all of the noise levels, respectively.

This result, again supports that the temporal information extracted from the first part of the model is meaningful, and more importantly, it shows that the decision threshold plays a role as it does in the behavior^10,25,26^. Importantly, Fig. 7B indicates that the decision bound in our model can be truly interpreted as the amount of evidence that the model needs to make a decision.

### Both accumulators truly accumulate information toward their preferred choices

Although Fig. 7B indicates that decision bounds set the speed-accuracy trade-off, it is not evident whether the bound of each accumulator can separately change the amount of information required for each decision in the model. Thus, we changed the decision bounds of each accumulator separately. Figure 7C, D indicate the probability of correctly detecting a face and a house in the stimulus with 26% of noise on different decision bounds, respectively. Based on Fig. 7C, D, performance of face (house) recognition increases as the bound of the face-accumulator (house-accumulator) increases. As a result, panel C and D in Fig. 7 show that both accumulators are integrating informative evidence about their preferred choices (Results are similar for other noise levels).

### The model shows similar behaviors for input images without artificial noise

**Figure 8:**
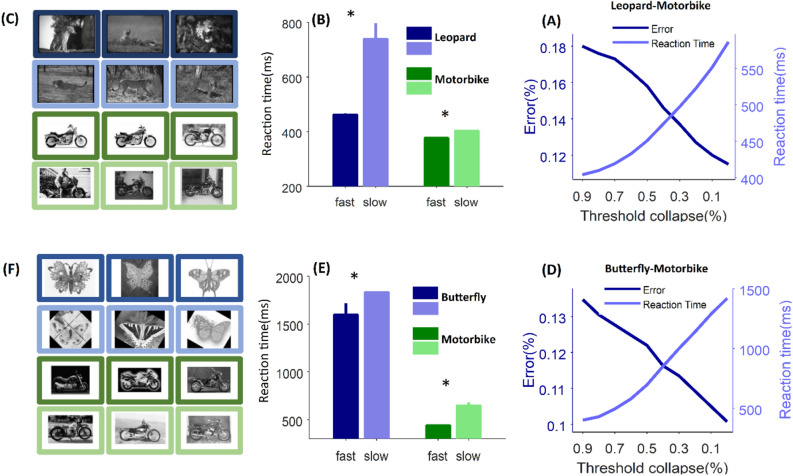
Model behavior on leopard-motorbike and butterfly-motorbike categorization. (**A**) Speed-accuracy trade off can be controlled by threshold on leopard-motorbike categorization. The X-axis indicates the percentage of collapse in the decision bound y axis shows error rate and reaction time of the model. (**B**) Average reaction time for the fast (dark colors) and the slow (bright colors) correct responses of leopard (blue) and motorbike (green) categories. Fast responses are 50% of trials which correctly touched the optimum threshold sooner and slow responses are the other half of trials which correctly touch the optimum threshold later. Here the optimum threshold is the threshold which minimize the error rate. (**C**) Samples of fast and slow responded images in both leopard and motorbike categories. Colors are consistent with those in the bar graph. (**D**), (**E**) and (**F**) are similar to (**A**), (**B**), (**C**) respectively but for the butterfly-motorbike categorization.

Aforementioned results showed that the model is capable of categorizing the input noisy stimuli with different speed and accuracy. However, the observed behavior in the model might be the result of the model sensitivity to the method we generate noisy images. In other words, the variety which is observed in the response times of the model might be a result of the different noise levels in the input image, not the difficulty of the content of the image (face or house). In order to examine this hypothesis, we tested the model with two other categorization tasks (leopard-motorbike and butterfly-motorbike) without inducing artificial noise in the input stimuli. As shown in Fig. 8A, D collapsing the decision bound from its optimum value (the optimum decision bound in this experiment is the value in which the model has the maximum categorization performance) to 90% of the optimal value increases the error rate (1-performance) while decreases the model reaction time in both leopard-motorbike and butterfly-motorbike tasks. This indicates that the decision bound still plays the role of speed-accuracy trade-off in categorization of stimulus without artificial noise. In addition to that, the model produces different reaction times when the decision bound is constant (Fig. 8B, E). In these figures, the decision bound is constant on its optimal value where the categorization accuracy is maximized. We sorted the reaction time of correct responses and called the first and the second half the “fast” and the “slow” responses, respectively. Bars in Fig. 8B, E show the average reaction times of the fast and the slow responses. There is always a significant difference between reaction times of fast and slow responses in both tasks (Mann–Whitney rank sum test, *P* < 0.03 for all categories). It should be noted that here we did not do a psychophysical experiment, thus we find the free parameters of the model in a way to maximize the accuracy and have the average reaction time below 2000 ms. Figure 8C, F show samples that the model categorized them very fast (dark blue and dark green) and very slow (light blue and light green) in both leopard-motorbike (Fig. 8C) and butterfly-motorbike (Fig. 8F) categorization tasks. These samples clearly show that the model responds slower when the stimulus contains a background or when the features of the target in the stimulus are visually less detectable compared to the fast responded images.

## Discussion and conclusion

Most of the studies which have tried to investigate the mechanisms of object recognition in the brain through computational modeling, have evaluated their proposed models by comparing the recognition accuracy of the model with that in human or animal^11,15–19^. Comparing the representation of input stimulus in the model with that in the brain is also another common way of model evaluation. However, both approaches are ignoring the time of recognition and the representation dynamics of the brain. In this study, we proposed a temporal model of human object recognition in both representation and decision levels. In the proposed model, by using a biologically plausible method, the input image is firstly represented temporally in the representation level (in layers preceding the decision-making layer); and then, in the decision-making level, the accumulated temporal evidence is used for making a final decision about the stimulus nature. The decision-making segment of the model is in fact a one-winner-take-all apparatus, comprising of two competing accumulators, which can finalize the decision as soon as the accumulated evidence for one option reaches a threshold. This accumulation-to-bound model is a well-known model that successfully explains most neurobiological and behavioral findings in decision-making studies^27,41–43^. The probability of correct choices increases if the decision layer of the model accumulates more information over time, indicating that the temporal evidence is not redundant. As a result, the model is able to explain not only the human choices in an object recognition task, but also the timing of those choices.

According to the ability of the proposed model to reproduce the performance and reaction times of human participants in an object recognition task, we can conclude that the suggested model is a good candidate for explaining the underlying mechanisms of object recognition in the brain. Considering this, the representation dynamics which stem from stimulus features and the decision bound which controls the speed and accuracy trade-off are two main factors causing variations in human reaction times when making a choice. Although these factors were discussed separately in different studies, we hypothesized that both should be considered to explain the time and the accuracy of object recognition in the human brain.

It is also important to note that the speed-accuracy trade-off in the model is not evident in all regimes of input information and decision threshold. As shown in Fig. 7, the choice will not be necessarily improved by collecting more information. In other words, the model shows that collecting noisy evidence may result in increasing the probability of touching the wrong bound. However, to the best of our knowledge, this is not evident in previous behavioral studies. We think that the lack of recurrent connections in our model results in such behavior.

Although the proposed model uses a simple accumulation-to-bound mechanism to decide about the input image, it performs better than spiking HMAX and deep neural networks when images get noisier. It is noticeable that all models were trained with noiseless images in their representation layers, except deep networks that were trained with ImageNet. One may speculate that the poor results of deep networks might be compensated if they were only trained on face and house images, and thus, they would be able to find better distinctive features of these two categories in the input images. Although this hypothesis needs more investigations, we think that this is not the case in our experiment because these models perform well and better than other models and even than human subjects in low noise images. This shows that they found good representative features for face and house, but those features are not robust enough to noise. Another hypothesis is that the strength of the proposed model in classifying noisy images stems from the fact that it uses the information about human response times in the decision layer to set the decision bound. We could not investigate this hypothesis by feeding the information of response times to other models because they do not have any mechanism in their training procedure to use response times. However, Fig. 7A shows that the threshold optimization, which uses human response times in its cost function, affects the performance only in two highest levels of noise. Thus, even without any optimization, a high threshold would cause a better performance in other noise levels in comparison to other models. It should be noted that although we implemented the dropout method in the transfer learning step of deep models, other regularization methods may improve the performance of these models.

Finally, we should emphasize that the importance of the proposed model is not that its performance is better than others, but is the temporal representation and the biologically plausible decision-making method. We believe that it will open a new window into investigations of the object recognition mechanisms in the brain, considering the time of stimulus presentation and response dynamics. Many theoretical challenges in explaining the process of object recognition, such as wherefore different objects need different processing times, what the different levels of categorizations are, or how objects with different visual variations (such as rotations, illuminations, and contrast) are recognized, can be revisited using the new approach introduced here.

## Supplementary Information

Supplementary Information 1.
