# Supplementary material for: A temporal hierarchical feedforward model explains both the time and the accuracy of object recognition
Source: Sci Rep. 2021 Mar 11;11:5640. doi: 10.1038/s41598-021-85198-2 (PMC7970968; doi:10.1038/s41598-021-85198-2)
Supplement: Supplementary file 1 — Supplementary Information 1. [file 41598_2021_85198_MOESM1_ESM.docx]

**Supplementary materials**

**A temporal hierarchical feedforward model explains both the time and the accuracy of object recognition**

Hamed Heidari-Gorji^1,2^, Reza Ebrahimpour^1,2*^, Sajjad Zabbah^2**^,

^1^ Faculty of Computer Engineering, Shahid Rajaee Teacher Training University, P.O. Box: 16785-163, Tehran, Iran.

^2^ School of Cognitive Sciences, Institute for Research in Fundamental Sciences (IPM), P.O. Box: 19395-5746, Tehran, Iran.

*Corresponding author. Tel.: +9821 22294035; fax: +9821 22280352. E-mail address: [ebrahimpour@ipm.ir](mailto:ebrahimpour@ipm.ir), [rebrahimpour@sru.ac.ir](mailto:rebrahimpour@sru.ac.ir)

**Co-Corresponding author. E-mail address: [s.zabbah@ipm.ir](mailto:s.zabbah@ipm.ir),

**Figure S1.** Detailed diagram of the proposed model.


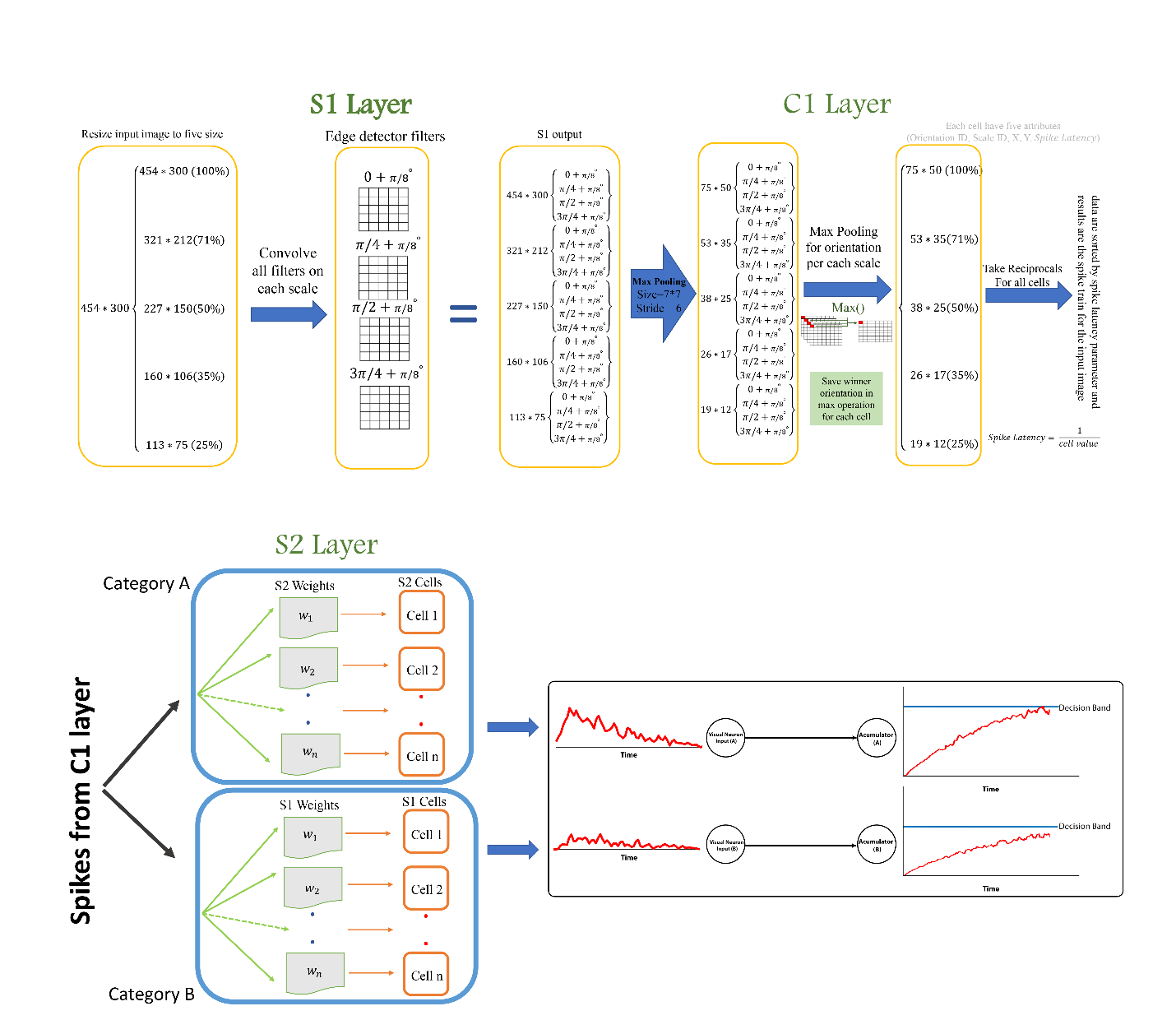


**Table S1.** Optimized parameters for decision making layer of the model

| Model time scale | Face threshold | House threshold | Non-decision time |
| --- | --- | --- | --- |
| 0.32 | 27.38 | 68.73 | 445.92 |
